# Impact of natural killer cells on outcomes after allogeneic hematopoietic stem cell transplantation: A systematic review and meta-analysis

**DOI:** 10.3389/fimmu.2022.1005031

**Published:** 2022-10-03

**Authors:** Muhammad Umair Mushtaq, Moazzam Shahzad, Amna Y. Shah, Sibgha Gull Chaudhary, Muhammad U. Zafar, Iqra Anwar, Karun Neupane, Ayesha Khalid, Nausheen Ahmed, Rajat Bansal, Ramesh Balusu, Anurag K. Singh, Sunil H. Abhyankar, Natalie S. Callander, Peiman Hematti, Joseph P. McGuirk

**Affiliations:** ^1^ Division of Hematologic Malignancies & Cellular Therapeutics, University of Kansas Medical Center, Kansas City, KS, United States; ^2^ Moffitt Cancer Center, University of South Florida, Tampa, FL, United States; ^3^ University of Wisconsin School of Medicine and Public Health, Madison, WI, United States

**Keywords:** allogeneic hematopoietic stem cell transplantation, immune reconstitution, hematologic malignancies, graft versus host disease, viral infections

## Abstract

**Background:**

Natural killer (NK) cells play a vital role in early immune reconstitution following allogeneic hematopoietic stem cell transplantation (HSCT).

**Methods:**

A literature search was performed on PubMed, Cochrane, and Clinical trials.gov through April 20, 2022. We included 21 studies reporting data on the impact of NK cells on outcomes after HSCT. Data was extracted following the PRISMA guidelines. Pooled analysis was done using the meta-package (Schwarzer et al.). Proportions with 95% confidence intervals (CI) were computed.

**Results:**

We included 1785 patients from 21 studies investigating the impact of NK cell reconstitution post-HSCT (8 studies/1455 patients), stem cell graft NK cell content (4 studies/185 patients), therapeutic NK cell infusions post-HSCT (5 studies/74 patients), and pre-emptive/prophylactic NK cell infusions post-HSCT (4 studies/77 patients). Higher NK cell reconstitution was associated with a better 2-year overall survival (OS) (high: 77%, 95%CI 0.73-0.82 vs low: 55%, 95%CI 0.37-0.72; n=899), however, pooled analysis for relapse rate (RR) or graft versus host disease (GVHD) could not be performed due to insufficient data. Higher graft NK cell content demonstrated a trend towards a better pooled OS (high: 65.2%, 95%CI 0.47-0.81 vs low: 46.5%, 95%CI 0.24-0.70; n=157), lower RR (high: 16.9%, 95%CI 0.10-0.25 vs low: 33%, 95%CI 0.04-0.72; n=157), and lower acute GVHD incidence (high: 27.6%, 95%CI 0.20-0.36 vs low: 49.7%, 95%CI 0.26-0.74; n=157). Therapeutic NK or cytokine-induced killer (CIK) cell infusions for hematologic relapse post-HSCT reported an overall response rate (ORR) and complete response (CR) of 48.9% and 11% with CIK cell infusions and 82.8% and 44.8% with NK cell infusions, respectively. RR, acute GVHD, and chronic GVHD were observed in 55.6% and 51.7%, 34.5% and 20%, and 20.7% and 11.1% of patients with CIK and NK cell infusions, respectively. Pre-emptive donor-derived NK cell infusions to prevent relapse post-HSCT had promising outcomes with 1-year OS of 69%, CR rate of 42%, ORR of 77%, RR of 28%, and acute and chronic GVHD rates of 24.9% and 3.7%, respectively.

**Conclusion:**

NK cells have a favorable impact on outcomes after HSCT. The optimal use of NK cell infusions post-HSCT may be in a pre-emptive fashion to prevent disease relapse.

## Introduction

Allogeneic hematopoietic stem cell transplantation (HSCT) is the optimal and potentially curative intervention for various high-risk hematologic malignancies. Allogeneic HSCT produces a graft versus leukemia (GVL) effect *via* an effective immune reconstitution in the recipient (1). Natural Killer (NK) cells are considered a part of the innate immune cells that constitute 5-15% of lymphocytes in the adult population (2). They possess significant antitumor and antiviral properties and donor-derived NK-cells are the first to reconstitute following HSCT (3).

Phenotypically, NK cells are defined as CD56^+^CD3^-^CD19^-^CD14^-^ mononuclear cells with the inclusion of alternative receptors such as natural cytotoxicity receptors (NCRs) (4). NK cell differentiation and maturation divide them into an immature CD56 bright phenotype that constitutes about 90% of peripheral blood NK cells and is responsible for an early cytotoxic immune response, and a mature CD56 dim phenotype that constitutes the remaining 10% of NK cells and lead to a delayed immune response by production of interferon-gamma (IFN-γ) and tumor necrosis factor (TNF) (5). The CD56 bright immature NK cell subset is the first to appear post-transplantation with the acquisition of mature CD56 dim phenotype months later (6). Several mechanisms have been described explaining the antitumor role of NK-cells following HSCT including their key role in orchestrating the anti-tumor immune response in the microenvironment, such as the production of proinflammatory cytokines (e.g. TNF-α, INF-γ, granulocyte colony-stimulating factor (G-CSF), etc.) and chemokines (chemokine (C-C motif) ligand 2-5 (CCL2-5), C-X-C motif chemokine ligand 8 (CXCL8), and chemokine C- motif ligand 1 (XCL1)), and an interplay between several inhibitory receptors such as receptors with intracytoplasmic signaling domains called immunoreceptor tyrosine-based inhibition motifs (ITIMs) and activating receptors such as NCRs and Natural Killer Group 2D (NKG2D) receptors (6–9).

Despite its remarkable potential, HSCT is associated with high relapse rates (RR) and significant non-relapse mortality (NRM). Efforts to potentiate the GVL effect, including increasing the conditioning intensity, reduction of post-transplant immune suppression, and donor lymphocyte infusions, have been limited by excessive treatment-related toxicity, high incidence of graft versus host disease (GVHD) requiring systemic immune suppression, and increased susceptibility to infections. NK cells have anti-neoplastic and anti-infective activity but do not cause alloreactivity and GVHD, and emerging data suggest the promising potential of NK cell therapy after HSCT (10–12). We hypothesized that higher reconstitution of NK cells following allogeneic HSCT, higher NK cell content of the stem cell graft, and pre-emptive or therapeutic use of NK cell infusions after allogeneic HSCT will result in improved outcomes after allogeneic HSCT, such as survival, relapse, GVHD, and infections.

## Methods

### Data sources and search strategy

This systematic review and meta-analysis was performed according to the Preferred Reporting Items for Systematic Reviews and Meta-Analysis (PRISMA) guidelines (13). Population, intervention, comparison, and outcome (PICO) table was developed and electronic databases of PubMed, Cochrane Library, and Clinical trial.gov were comprehensively and systematically searched by two authors (M.S., A.Y.S.) independently through April 20, 2022, using the following search terms: “hematologic malignancies” OR “hematopoietic stem cell transplantation” AND ”natural killer cells”. No filters or publication time limits were applied to the search. We also searched conference abstracts, including the American Society of Hematology and the American Society of Clinical Oncology. Our search identified 989 articles. All search results were imported to the Endnote X9.0 reference manager and duplicates were removed.

### Screening and selection criteria

**Figure 1:**
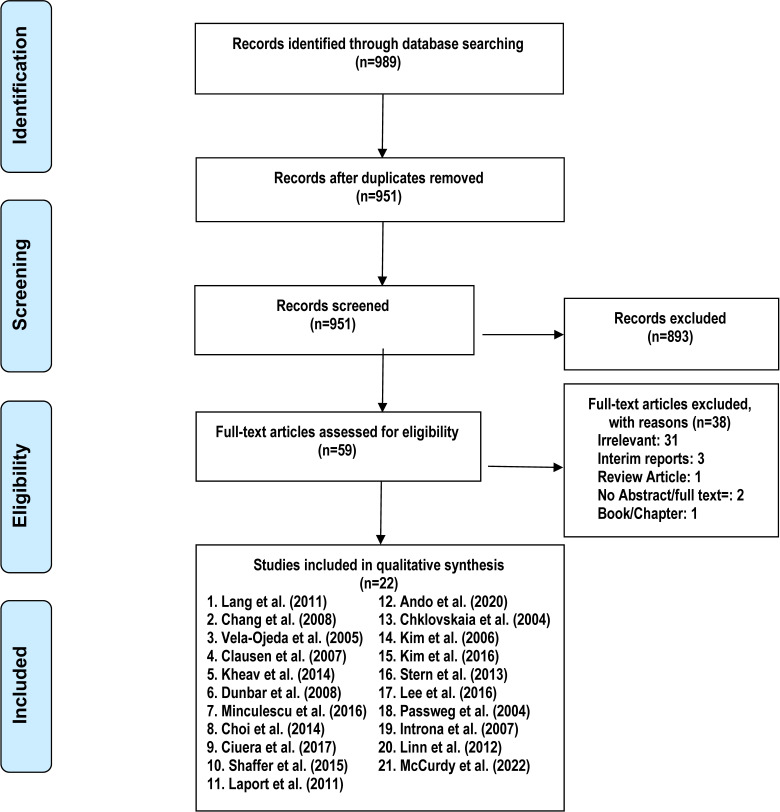
PRISMA diagram of included and excluded studies.

A title or abstract-specific primary screening was conducted by two authors (M.U.Z., A.Y.S.) independently to exclude irrelevant articles. Disagreements were resolved by mutual consensus and by a third author (M.S.). Full texts of the remaining 59 articles were then assessed for eligibility based on predetermined criteria which were set after discussion and consensus between all authors and approved by the PI (M.U.M.). Inclusion criteria were original studies (clinical trials retrospective, and prospective cohort), 2) both adult and pediatric patients, 3) studies that enrolled only allogeneic HSCT patients, 4) studies that investigated the impact of NK cells reconstitution after HSCT, graft NK cell content, or NK cell infusion after HSCT. We excluded reviews, studies with insufficient data, animal models, or studies presenting outcomes that were not relevant to the searching protocol. Articles not in English were excluded if translations were not available. A total of 38 articles were excluded in secondary screening based on case reports, case series, reviews, and/or irrelevant articles **(** **Figure 1** **) (** **Supplementary Table 1** **)**.

### Data extraction

An excel sheet was created for data extraction with consensus and approved by the PI (M.U.M.). Two authors (M.U.Z. and I.A.) extracted data independently. Data were extracted regarding author name, year of publication, the number of evaluable patients, age, gender, underlying disease, donor type, graft source, conditioning regimen, follow-up time, overall survival (OS), overall response rate (ORR), complete remission/response (CR), GVHD, RR, cytomegalovirus (CMV) reactivation, and NK cell donor, cell dose and conditioning (when applicable). In studies that reported dichotomous data, the results were analyzed by using high and low NK cell cut-offs per the individual studies. In the case of time-to-event analysis, we calculated the median OS for the most recurring timeperiod. Datasheets were double-checked by M.S. and M.U.M. for any discrepancies.

### Quality assessment

The methodological quality of the included studies was evaluated using the National Institute of Health (NIH) quality assessment tool for before-after (pre-post) studies with no control group. Based on this tool, the studies were rated as good and fair.

### Data analysis

Meta-analyses were performed using R-studio. The ‘metaprop’ package (Schwarzer et al, R programming language) was used to calculate the pooled risks using the random effects model. For calculating inter-study variance, Der Simonian-Laird Estimator was used. We assessed heterogeneity in the meta-analysis with the *I* (2) statistics (14). The test uses χ (2) and degrees of freedom to describe the percentage of the variability in effect estimates that is due to heterogeneity rather than sampling error (chance) (13). *I* (2) reflects the percentage of total variation across studies and values greater than 25%, 50%, or 75% were considered to respectively indicate low, moderate, or high heterogeneity (15). If *p*<0.05, the pooled analysis was considered significantly heterogeneous (13). τ (2) was used to estimate the dispersion of true effect sizes between studies, with low values meaning low dispersion and consequently low heterogeneity (13).

## Results

We included a total of 1785 patients from 21 studies.

### Reconstitution of NK cells after HSCT

A total of 1455 patients from 8 studies investigating the impact of NK cell reconstitution following allogeneic HSCT on post-transplant outcomes were evaluated (16–23) **(** **Table 1** **)**. The median age of the patients was 43 (8.6-73) years and 37% were male. The median duration of follow-up was 32 (1.4-122) months. The graft source was peripheral blood (PB) for 47.5% (691/1455), bone marrow (BM) for 44% (642/1455), and umbilical cord blood (UCB) for 8.3% (122/1455) of the patients. The donor type was matched sibling donor (MSD) 33.5% (406/1209), matched unrelated donor (MUD) 47.5% (575/1209), haploidentical (haplo) 12.9% (156/1209), and mismatched unrelated donor (MMUD) 5% (62/1209). Myeloablative conditioning (MAC) was used in 50% (734/1455) of the patients and 46% (676/1455) of patients received reduced-intensity conditioning (RIC). The hematologic diagnoses were acute lymphocytic leukemia (ALL) 14.6% (213/1143), acute myeloid leukemia (AML) 39.9% (457/1143), myelodysplastic syndromes (MDS) 16.2% (186/1143), chronic myeloid leukemia (CML) 2.6% (30/1143), lymphoma (Hodgkin and non-Hodgkin) 6.5% (74/1143), myeloproliferative neoplasm (MPN) 4.1% (47/1143), multiple myeloma (MM) 3.9% (45/1143), and others 7.9% (91/1143). NK cells were grouped into high and low by using existing criteria from individual studies.

**Table 1:** Impact of NK cells reconstitution on outcomes after allogeneic HSCT.

Study	Pts, n	Male, n (%)	Age (yrs), Median (range)	Donor Type, n (%)	Graft source, n (%)	Diagnosis, n (%)	Conditioning, n (%)	GVHD Prophylaxis, n (%); T- cell Depletion	Follow up, median (range) months	Acute GVHD, n (%), Grade	Relapse, n (%)	CMV reactivation, n (%)	OS, (%) years (Y)
Lang et al.^a^ (2011) (18)	47	NA	8.6	MUD 18 (38) Haplo 29 (61)	BM 47 (100)	ALL 27 (57), AML 10 (21), MDS 2 (4.2), CML 4 (8.5), JMML 1 (2), Lymphoma 3 (6)	MAC 47 (100)	NR; yes	NA	NA	G1:11 (10), G2: 5.5 (25), G3: 4.5 (50), G4: 0 (0)	NR	NA
Ando et al.^b^ (2020) (19)	246	152 (61.8)	51 (18-69)	NA	BM 98 (39.8) PB 36 (14.6) UCB 112 (45.5)	AML 131 (53.4), ALL 61 (24.8), MDS 32(13), CML 7 (2.8), MPN 7 (2.8), other 8(3.2)	MAC 99 (40.2), RIC 147(59.8)	Tac, CSA; no	38.4 (9.6-115)	98 (40), >II	NA	141 (57)	High: 75 Low: 35.7 at 5Y
Chang et al.^c^ (2008) (20)	43	29 (67)	27 (13-40)	Haplo 43 (100)	PB 43 (100)	AML 14 (33), ALL 12 (28), CML 16 (37), MDS 1 (2)	MAC 43 (100)	CSA, MTX, MMF; yes	12 (1.4 –23)	25 (58), >II	High:0 (0) Low: 3 (21)	NR	High: 92.9, Middle: 66.7 Low: 42.9 at 2Y
Kheav et al. (2014) (21)	439	NA	44 (15-68)	MSD 237 (54) MUD 151 (33.9) MMUD 51(12)	PB 336 (76.5) BM103 (23.5)	AML 104 (23.7) ALL 57 (13) NHL 49 (11.2), HL 19 (4.3), MM 42 (9.6), MPN 40 (9.1), MDS 52 (11.8), other 76 (17)	RIC 266 (60.6) MAC 173 (39.4)	NR; no	NA	205 (48), >II	89 (22)	269 (61.5)	NA
Dunbar et al.^d^ (2008) (22)	167	Low: 39 (47) High:16 (56)	Low: 44 High: 48	MSD/MUD 149 (89) UCB 10 (5.9) single HLA-MM 8 (4.7)	PB 157(94) UCB 10(5.9)	NA	MAC 79 (47) RIC 43(25)	Tac; MMF; no	12.3 (2.2-58)	High: 13 (34) Low: 40 (34), I	HR: 20.2 (Low vs high)	NR	High: 72 Low: 83 at 1 Y
Kim et al.^e^ (2016) (23)	70	34 (49)	42 (20-64)	MSD 34 (48.5) Haplo 6 (8.5) MUD 19 (2.7) MMUD 11 (15.7)	PB 59 (84) BM 11 (16)	ALL 14 (20), AML 33 (47), CML 3 (4.3) MDS 8 (11) MM 3 (4.3), lymphoma 3 (4.3) other 6 (8.6)	MAC 41 (59) RIC 29 (41)	NA; yes	32.8	35 (50), >I	21 (30)	NR	High: HR 1, Low: HR 2.24 1.04-4.83) at 1 Y
Minculescu et al.^f^ (2016) (17)	298 NK<150: 102 NK>150: 196	173 (58) NK <150: 37 (36) NK >150: 88 (44.8)	55 (16-74) NK <150 53 (16-74) NK >150 55 (18-73)	MSD 100 (33.5) MUD 198 (66)	PB 60 (20) BM 238 (79.8)	ALL 42 (14), AML 165 (55), MDS 91 (30.5)	MAC 107 (35.9) RIC 191 (64)	MAC: CSA, MTX and RIC: Tac, MMF; no Non-MAC: Tacrolimus +MMF; no	47.9 (15.6-122)	127 (43), > I	64 (21)	High: 37 (19) Low: 35 (34)	High: 76 Low: 61.8 at 1 Y
McCurdy et al^g^ (2022) (16)	145	MSD/MUD 55 (73) Haplo 46 (65)	MSD/MUD 49 (22-64) Haplo 41 (2-64)	MSD 35 (24) MUD 40 (28) Haplo 70 (48)	BM 145 (100)	NA	MAC 145 (100)	PTCy, Tac, MMF; no	NR	II-IV 55 (38) III-IV 16 (11)	High: 21 Low: 35 at 2 Y	MSD/MUD 39 (53) Haplo 28 (40)	High: 81 Low: 50 at 2 Y

a Groups were divided based on the patterns of NK activity. Group 1 (consistently low activity <10% specific lysis), group 2 (consistently high activity >30% specific lysis), group 3 (initially high activity then decreasing), group 4 (initially low activity, then increasing to normal levels).

b Not mentioned.

c High (>9.27 cells/μL, n=14), Middle (3.59 to 9.27 cells/μL, n=51), Low (<3.59 cells/μL, n=14).

d ANK levels were designated from the cluster analysis and counts greater than 18.2 cells/mm3 at 60 days were considered in the high level (n=84) and counts less than this value was considered in the low level (n=38).

e Cut-off values were different for 30 days vs 90 days post-transplant. For 30 days post-transplant the cut-off was <177.5/μL. For 90 days post-transplant cut-off was <181.9 cells/μL. OS was HR=1 at High NK and HR=2.64(0.76-9.21) at Low NK.

f High: NK30>150, Low: NK30<150.

g High NK cell count: >50.5 cells/mL and Low NK cell count: £50.5 cells/mL

OS, overall survival; NA, not available; ALL, acute lymphoid leukemia; AML, acute myeloid leukemia; BM, bone marrow; UCB, umbilical cord blood; CLL, chronic lymphoid leukemia; HL, Hodgkin lymphoma; JMML, juvenile myelomonocytic leukemia; MAC, myeloablative conditioning; MDS, myelodysplastic syndrome; MUD, matched unrelated donor; MSD, matched sibling donor; Haplo-haploidentical donor; MMUD, mismatched unrelated donor; MM, multiple myeloma; NHL, non-Hodgkin lymphoma; NR, nonreported; PB, peripheral blood; RIC, reduced intensity conditioning; SAA, severe aplastic anemia; MPN, myeloproliferative neoplasm; TBI, total body irradiation; TCD, T-cell depleted; HR, hazard ratio; NMA, nonmyeloablative; AA, aplastic anemia; CSA, cyclosporin; Tac, tacrolimus; MMF, mycophenolate; MTX, methotrexate; PTCy, post-transplant cyclophosphamide.

**Figure 2:**
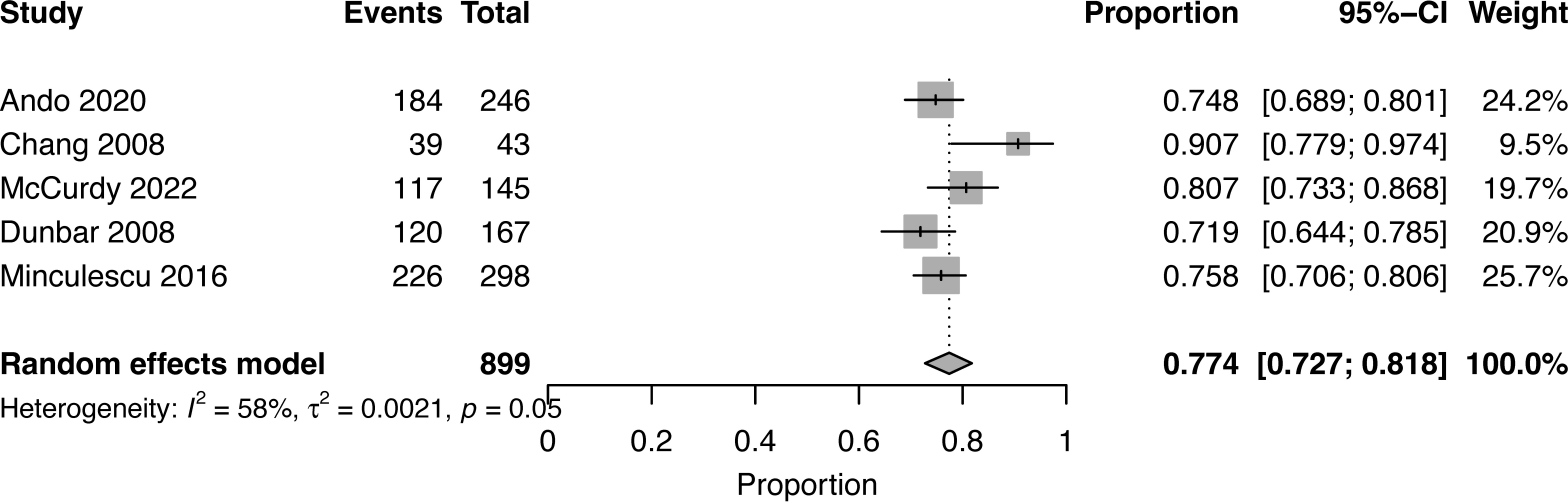
Forest plot of overall survival for high NK cells reconstitution after allogeneic HSCT.

**Figure 3:**
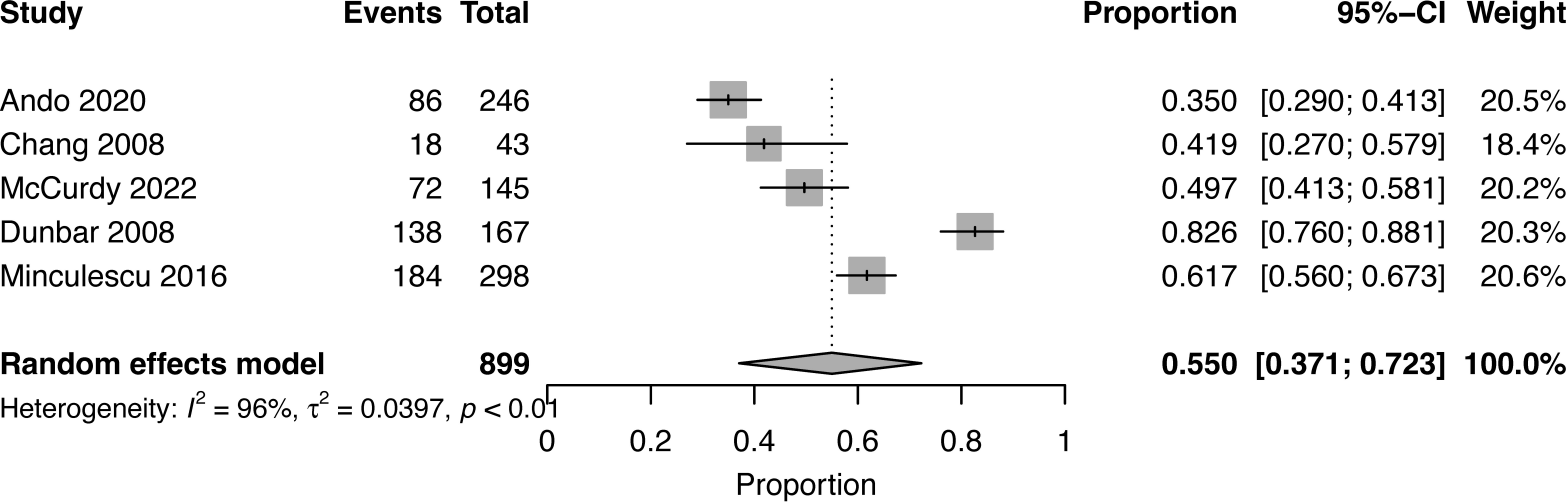
Forest plot of overall survival for low NK cells reconstitution after allogeneic HSCT.

At a median follow-up of 2 (1-5) years, the pooled OS for high NK cell cohort and low NK cohort was 77% (95% CI 0.73-0.82, I^2 =^ 58%, n=899) and 55% (95% CI 0.37-0.72, I^2 =^ 96%, n=899), respectively (16, 17, 19, 20, 22) **(** **Figures 2**, **3** **)**. The pooled RR was 30% (95% CI 0.18-0.43, I^2 =^ 94%, n=952) (17, 21, 23). The pooled incidence of acute GVHD was 59% (95% CI 0.44-0.74, I^2 =^ 96%, n=1241) (16, 17, 19–21, 23). The incidence of CMV infections was 56% (95% CI 0.47-0.63, I^2 =^ 80%, n=830) (16, 19, 21). Minculescu et al. reported that higher NK cell reconstitution was significantly associated with lower CMV infections (19% vs 34%) (17). Dunbar et al. reported higher RR (adjusted hazard ratio 20.2) with low NK cell count at 60 days post-HSCT and there was no significant association with GVHD incidence (22). Lang et al. reported significantly lower RR with higher NK cell reconstitution (18% vs 73% at 2 years) (18). McCurdy et al. recently reported a significantly lower RR (21% vs 35% at 2 years), lower NRM (4% vs 21%), and higher 2-year OS and PFS (81% and 76% vs 50% and 44%, respectively) with higher NK cell constitution using a cut off of >50.5 NK cells/µL at day 28 post-HSCT (16). The data were insufficient to conduct a pooled analysis for relapse, GVHD, and CMV infections.

### Stem cell graft NK cell content

A total of 185 patients from 4 studies investigating the impact of NK cell content of stem cell graft on post-transplant outcomes were evaluated (24–27) (**Table 2** **)**. The median age was 35 (16-73) years and 32% were male. In three of four included studies with available data (n=157/185), all patients received a matched sibling donor peripheral blood stem cell transplantation. Most patients (80%, 148/185) received a myeloablative conditioning regimen. The hematological diagnoses were AML/ALL 56.7% (105/185), CML 25.9% (48/185), lymphoma 7% (13/185), MDS 4.8% (9/185), MM 3.2% (6/185), and severe aplastic anemia 1.6% (3/185).

**Table 2:** Impact of graft cell content of NK cells on outcomes after allogeneic HSCT.

Study	Pts, n	Male, n (%)	Age (yrs), Median (range)	Donor Type, n (%)	Graft source, n (%)	Diagnosis, n (%)	Conditioning, n (%)	Follow up, median (range) months	Acute GVHD, n (%), Grade	Relapse, n (%)	CMV reactivation, n (%)	OS, n (%) years (Y)
Chklovskaia et al. (2004) (27)	28	NA	32 (16 – 51)	NA	PB 22 (78) BM 6 (21)	AML 9 (32) CML 10 (35.7), MDS 2 (7), MM 1 (3.5), SAA 3 (10.7), ALL 3 (10.7)	MAC 28 (100)	NA	8 (28.5), 0-I 14 (50), >II	NA	NA	NA
Vela-Ojeda et al. (2005)^a^	45	32 (71)	34 (16 - 53)	MSD 45 (100)	PB 45 (100)	AML 9 (20), ALL 9 (20), CML 24 (53), MM 2 (4), NHL 1 (3)	MAC 45 (100)	18 (6 – 38)	9 (21), >I	5 (11)	NA	High: 0.5 at 1.5Y Low: 0.7 at 2.3Y
Kim et al. (2006)^b^ (26)	69	28 (40.5)	36 (17 – 58)	MSD 69 (100)	PB 69 (100)	AML 41(59), ALL 9(13), CML 8 (12), MDS 2(3), Lymphoma 9(13)	MAC 51 (74), RIC 18 (26)	13.4 (0.3 – 77.6)	≥50^th^: 19 (70) <50^th^: 26 (90), >II	≥50^th^: 12 (41) <50^th^: 11 (38)	NA	≥50^th^ percentile: 64.9% ± 9.0, <50^th^ percentile: 32.1% ± 8.9 at 2Y
Clausen et al. (2007)^c^ (25)	43	NA	43 (17 – 73)	MSD 43 (100)	PB 43 (100)	ALL+AML 25 (58), CML 6 (14), MDS 5 (12), NHL 3 (7), MM 3 (7), other 1 (2)	MAC 24 (55.8), RIC 19 (44)	17.4 (1.6 – 46.8)	High: 12 (27) Low: 27 (62), >II	High: 7 (16) Low: 23 (54)	High: 7 (17) Low: 27 (63)	High: 83% Low: 37% at 2Y

a Median cut- of 3 x107 for high and low NK.

b ≥50^th^ percentile: n = 29, <50^th^ percentile: n = 29, based on Log rank test.

c The threshold between ‘low’ and ‘high’ graft cell counts was set according to the respective median values for NK cells (x108/kg) 0·32 for entire cohort, while 0.34 for standard intensity and 0.31 for RIC.

HSCT, hematopoietic stem cell transplant; CR, complete remission; ORR, Overall response rate; NA, Not available; AML, Acute myeloid leukemia; ALL, Acute lymphoblastic leukemia; HD, Hodgkin’s lymphoma; SCA, Sarcoma; MDS,Myelodysplastic syndrome; NHL, Non-Hodgkin’s lymphoma; CML, Chronic myeloid leukemia; CMML, Chronic myelomonocytic leukemia; MM, Multiple myeloma; CLL, Chronic lymphocytic leukemia; Haplo, Haploidentical; MRD, Matched related donor: MUD, Matched unrelated donor; MAC, Myeloablative conditioning: TCD, T cell depleted; RIC, reduced intensity conditioning; CIK cells, Cytokine-induced killer cells; PB, peripheral blood.

At the median follow-up of 17.4 (0.3-77.6) months, the pooled OS was 65.2% (95% CI 0.471-0.814, I^2 =^ 81%, n=157) for high NK cell group and 46.5% (95% CI 0.235-0.704, I^2 =^ 89%, n=157) for low NK cell group (24–26). The pooled RR was 16.9% (95% CI 0.104-0.246, I^2 =^ 0, n=157) for high NK cell group and 33% (95% CI 0.040-0.721, I^2 =^ 94%, n=157) low NK cell group (25, 26). The pooled incidence of acute GVHD was 27.6% (95% CI 0.196-0.364, I^2 =^ 0, n=157) for high NK cell group and 49.7% (95% CI 0.259-0.737, I^2 =^ 85%, n=157) for low NK cell group (25, 26).

### Therapeutic use of NK cells for hematologic relapse after HSCT

A total of 74 patients from 5 studies were evaluated that investigated the use of NK cells for the relapsed disease after allogeneic HSCT (28–32) **(** **Table 3** **)**. The median age of patients was 42.4 (1.9-69) years and 36.5% were male. The median duration of follow-up was 20 (1-69) months. The donor type was MSD 59.4% (44/74), MUD 22.9% (17/74), haplo 55% (41/74), and UCB 1.3% (1/74). Conditioning chemotherapy (fludarabine/cyclophosphamide) before NK cell infusion was used in two studies (31, 32). Other three studies did not use any conditioning; however, concurrent investigator’s choice therapy was allowed (28–30). The underlying diagnoses were ALL 8% (6/74), AML 39% (29/74), lymphoma 17.5% (13/74), chronic lymphocytic leukemia (CLL) 2.7% (2/74), CML 12% (9/74), MM 4% (3/74), and MDS 16% (12/74). All five studies reported varying NK cell infusion dosages, with a range of 1 x10 (6) NK cells/kg to 1 x10 (8) NK cells/kg (28–32).

**Table 3:** Impact of therapeutic use of NK and CIK cell infusions on outcomes after allogeneic HSCT.

Author/Year	Shaffer et al., 2015	Lee et al., 2016	Introna et al., 2007	Laport et al., 2011	Linn et al., 2012
**Study type**	Clinical Trial Phase 2, NK cells	Clinical Trial Phase 1	Clinical Trial Phase 1, CIK cells	Clinical Trial Phase 1, CIK cells	Clinical Trial Phase 1-2, CIK cells
**Patients, n**	8	21	11	18	16
**Age (years), Median (range)**	19 (1.9-55.9)	51 (2-63)	53 (24-62)	53 (20-69)	36 (20-60)
**Male, n (%)**	NA	15 (71)	5 (45.4)	NA	7 (44)
**Diagnosis, n (%)**	AML: 6 (75), MDS: 2 (25)	AML: 8 (38), CML: 7 (30), MDS: 6 (28.5)	AML: 4 (36), HD: 3 (27), CML: 1 (9), ALL: 1(9), MDS: 2 (18)	NHL: 5 (27), AML: 3 (16), MM: 3 (16), CLL: 2 (10), ALL: 2 (10), MDS: 2 (10), HL: 1 (5)	ALL: 3 (18), HD: 3 (18), AML: 8 (50), CML: 1 (6), NHL: 1 (6)
**Disease status**	Relapsed or persistent disease	Relapsed or persistent disease	Relapsed disease	Relapsed disease	Relapsed disease
**HSCT donor**	MSD: 7 (87.5); MUD: 1(12.5)	MSD 13 (72%); MUD 8 (38%)	MSD: 6 (54); MUD: 5 (45)	MSD: 18 (100)	Haplo: 12 (75); MUD: 3 (19); UCB: 1(6)
**NK/CIK cell donor**	Haplo: 8 (100). Third party	Haplo: 21 (100), third party	Autologous CIK cells: 11 (100)	MSD: 18 (100). Same matched sibling donor	HSCT donors: 20; Autologous: 5^a^
**Product Manipulation**	PBMCs collected by apheresis, CD3+ T-cell depletion with CliniMACS, followed by CD56+ selection	PBMCs collected by apheresis, CD3+ T-cell depletion with CliniMACS (first 3 patients received CD56+ selection), culture with IL-2; post-infusion IL-2	Leukapheresis product was expanded using CIK cells expansion cultures, using IFN-γ, CD3 antibody, and IL-2	Leukapheresis product was expanded using CIK cells expansion cultures, using IFN-γ, CD3 antibody, and IL-2	Leukapheresis product was expanded using CIK cells expansion cultures, using IFN-γ, CD3 antibody, and IL-2
**Number of infusions**	1	4	Repeated infusions at 3–4-week intervals; Median number of CIK infusions were 2	1	55 (1–12 infusions per patient); at a minimum of 4-week intervals
**NK cell infusion dose/kg, (range)**	10.6 (4.3 - 22.4) x 10^6^	Escalating dosage: 1 x 10^6^; 5 x 10^6^; 3 x 10^7^; 3 x 10^7^	12.4 (7.2 - 87.4) x 10^6^	Escalating dose: 1 x 10^7^; 5 x 10^7^; 1 x 10^8^	10-200 x 10^6^
**Pre-NK cell Conditioning**	Flu/Cy with IL-2	RIC (BuFluATG) 21 (100)	None. Other therapies included DLI 3, Chemotherapy 2, DLI plus Chemo 3, RT 1	None	None. Treated with investigator’s choice therapy concurrently
**CR, n (%)**	2 (25)	11 (78.5)	3 (27.2)	2 (11.1)	NA
**ORR, n (%)**	3 (37.5)	21 (100)	3 (27.2)	14 (77.7)	5 (31.2)
**OS, n (%)**	NA	5 (23%) – 7.6 months	4 (36.3%); Median OS 2.5 (2.1-2.7) years	10 (55.5%) at 2.3 years; Median OS 28 months	NA^b^
**Follow up (months), median (range)**	NA	NA	20.5	20 (1-69)	4
**Relapse, n (%)**	3 (37.5)	12 (57.1)	NA	16 (88.8)	9 (56.2)
**Acute GVHD, n (%)**	0	10 (47.6)	4 (36.3)	2 (11.1)	3 (18.7)
**Chronic GVHD, n (%)**	0	6 (30)	2 (18.1)	1 (5.5)	2 (12.5)

a Donor cells may be mobilized peripheral blood stem cells (cryopreserved), leukapheresis product or harvested from patient if donor not available

b Individual patient level data available, cumulative data not available.

HSCT, hematopoietic stem cell transplant; CR, complete remission; ORR, Overall response rate; NA, Not available; AML, Acute myeloid leukemia; ALL, Acute lymphoblastic leukemia; HD, Hodgkin’s lymphoma; SCA, Sarcoma; MDS,Myelodysplastic syndrome; NHL, Non-Hodgkin’s lymphoma; CML, Chronic myeloid leukemia; CMML, Chronic myelomonocytic leukemia; MM, Multiple myeloma; CLL, Chronic lymphocytic leukemia; UCB – Umbilical Cord Blood; Haplo, Haploidentical; MSD, Matched sibling donor: MUD, Matched unrelated donor; MAC, Myeloablative conditioning: TCD, T cell depleted; RIC, reduced intensity conditioning; BuFluATG, Busulfan, fludarabine, thymoglobulin; PT-Cy, Post-transplant cyclophosphamide; FluCy, Fludarabine, cyclophosphamide; CIK cells, Cytokine-induced killer cells; PBMCs, peripheral blood mononuclear cells; IFN-γ, Interferon-gamma; IL, Interleukin

Two studies, including 29 patients, used third-party haplo NK cells. Peripheral blood mononuclear cells (PBMCs) were collected by apheresis followed by CD3+ depletion and CD56+ selection with CliniMACS (31, 32). ORR and CR were noted in 82.8% (24/29) and 44.8% (13/29) of the patients, respectively. RR was 51.7% (15/29). Median OS was reported as 7.6 months by Lee et al. Acute and chronic GVHD occurred in 34.5% (10/29) and 20.7% (6/29) of the patients, respectively. Three studies, including 45 patients, used donor-derived or autologous cytokine-induced killer (CIK) cells. Leukapheresis product was expanded using CIK cells expansion cultures, using interferon-gamma (IFN-γ), CD3 monoclonal antibody, and interleukin (IL)-2 (28–30). ORR and CR rates were reported as 48.9% (22/45) and 11.1% (5/45). Median OS was reported as 2.4 years by two studies. RR was 55.6% (25/45). Acute and chronic GVHD was noted in 20% (9/45) and 11.1% (5/45) of the patients, respectively.

### Pre-emptive use of NK cells to prevent relapse after HSCT

A total of 77 patients from 4 studies were evaluated that investigated the pre-emptive (or prophylactic) use of NK cells to prevent disease relapse after allogeneic HSCT (33–36) **(** **Table 4** **)**. The median age of patients was 33.5 (2-75) years and 40% were male. The median duration of follow-up was 23.1 (8-81.6) months. All four studies used haplo donors. RIC was used in 70% (54/77) of the patients and MAC was used in 27% (21/77) of the patients. The hematologic diagnoses were ALL 15.5% (12/77), AML 67.5% (52/77), lymphoma 3.8% (3/77), MDS 2.6% (2/77), CML 7.7% (6/77), and sarcoma 1% (1/77). All four studies used donor-derived NK cells. PBMCs were collected by apheresis followed by CD3+ depletion and CD56+ selection with CliniMACS. Recently conducted studies by Choi et al. and Ciuera et al. also performed ex vivo expansion as detailed in **Table 4**. Patients received 2-3 NK cell infusions. NK cell infusion doses ranged from 1 x10 (5) cells/kg to 1 x10 (8) cells/kg (33–36).

**Table 4:** Impact of pre-emptive/prophylactic NK cells infusions on outcomes after haploidentical HSCT.

Author/Year	Stern et al., 2013	Choi et al., 2014	Ciuera et al., 2017	Passweg et al., 2004
**Study type**	Clinical Trial Phase 2	Clinical Trial Phase 1	Clinical Trial Phase 1	Pilot Study
**Patients, n**	16	41	13	5
**Age (years), Median (range)**	A: 23 (8-32), B: 10 (8-23)	47 (17-75)	44 (18-60)	16 (3-25)
**Male, n (%)**	NA	23 (56)	6 (46)	2 (40)
**Diagnosis, n (%)**	AML: 8 (50), ALL: 5 (31), HL: 2 (12.5), SCA: 1 (6)	ALL: 7 (17), AML: 32 (78), MDS: 1 (2), NHL: 1 (2)	AML: 8 (61.5), CML: 5 (38), MDS: 1 (7.7)	AML: 4 (80), CML: 1 (20)
Diagnosis specification	Haplo HSCT	Haplo HSCT	High risk myeloid malignancies	NK-DLI in case of poor engraftment or relapse
**HSCT donor**	Haplo: 16 (100)	Haplo: 41 (100)	Haplo: 13 (100)	Haplo: 5 (100)
**NK cell donor**	Same as HSCT donor	Same as HSCT donor	Same as HSCT donor	Same as HSCT donor
**Product Manipulation**	PBMCs collected by apheresis, CD3+ T-cell depletion with CliniMACS, followed by CD56+ selection	PBMCs collected by apheresis, CD3+ T-cell depletion with CliniMACS or RosetteSep system. Cultured with IL-15, IL-21 and hydrocortisone	PBMCs collected by apheresis, CD3+ T-cell depletion with CliniMACS. Ex vivo expansion using K562 feeder cells expressing membrane-bound IL-21	PBMCs collected by apheresis, CD3+ T-cell depletion with CliniMACS, followed by CD56+ selection
**Timing of infusion**	Center A: Day +40, +100 Center B: Day +3, +40, +100	First infusion 2 weeks after HCT, second infusion 3 weeks after HCT	Day +2, +7, +28	NK-DLI in case of poor engraftment (n = 3), graft failure (n = 1), and early relapse (n = 1)
**Number of infusions**	29 total, 1.8 per patient	2	3	2
**NK cell infusion dose/kg, (range)**	1.21 (0.3-3.8) x10^7^	Escalating dosage: 0.2 x 10^8^; 0.5 x 10^8^; 1.0 x 10^8^; ≥1.0 x 10^8^	1 x 10^5^ – 1 x 10^8^	1.61 (0.21-2.2) x 10^7^
**HSCT Conditioning**	MAC/TCD 16 (100)	RIC (BuFluATG) 41 (100)	Mel based RIC with PT-Cy: 13 (100)	MAC/TCD: 5 (100)
**CR, n (%)**	2 (12.5)	AML: 21 (72); ALL/NHL: 4 (50)	NA	3 (60)
**ORR, n (%)**	13 (81.3)	NA	NA	3 (60)
**OS, n (%)**	44 ± 12% at 1 yr: 25 ± 11% - 2 & 5 yrs	AML: 31% -4 yrs; ALL/NHL:0 -1 yrs	11 (85%) – 1 yr	4 (80%)- 1 yr
**Follow up (months), median (range)**	69.6 (63.6-81.6)	31.5 (16-53)	14.7 (8-25.1)	12 (8-18)
**Relapse, n (%)**	7 (43.8)	15 (37)	1 (7.7)	1(20)
**Acute GVHD, n (%)**	4 (25)	9 (22)	7 (54)	0
**Chronic GVHD, n (%)**	0	10 (24)	0	0

HSCT, hematopoietic stem cell transplant; CR, complete remission; ORR, Overall response rate; NA, Not available; AML, Acute myeloid leukemia; ALL, Acute lymphoblastic leukemia; HD, Hodgkin’s lymphoma; SCA, Sarcoma; MDS,Myelodysplastic syndrome; NHL, Non-Hodgkin’s lymphoma; CML, Chronic myeloid leukemia; CMML, Chronic myelomonocytic leukemia; MM, Multiple myeloma; CLL, Chronic lymphocytic leukemia; Haplo, Haploidentical; MAC, Myeloablative conditioning: TCD, T cell depleted; RIC, reduced intensity conditioning; BuFluATG, Busulfan, fludarabine, thymoglobulin; PT-Cy, Post-transplant cyclophosphamide; FluCy, Fludarabine, cyclophosphamide; Mel, Melphalan; PBMCs, peripheral blood mononuclear cells; DLI, donor lymphocyte infusion; IL, Interleukin.

At the median follow-up of 1 (0.63-5) year, the pooled OS was 69% (95% CI 0.38-0.93, I^2 =^ 63%, n=34) (33, 34, 36). The pooled ORR was 77% (95% CI 0.55-0.94, I^2 =^ 0, n=21) (33, 34). The pooled CR was 42% (95% CI 0.08-0.79, I^2 =^ 84%, n=62) (33–35). The pooled RR was 28% (95% CI 0.13-0.46, I^2 =^ 47%, n=24) (33–36). The pooled incidence of acute and chronic GVHD was 24.9% (95% CI 0.08-0.44, I^2 =^ 57%, n=75) and 3.7% (95% CI 0.00-0.22, I^2 =^ 74%, n=10), respectively (33–36).

## Discussion

NK cells are cytotoxic lymphocytes and are an important part of the innate immune systems. They play an important role in early immune reconstitution after HSCT. NK cells are important mediators of anti-tumor and anti-infective responses in the recipient. In this systemic review and meta-analysis, we investigated the impact of NK cell reconstitution post-HSCT, stem cell graft NK cell content, therapeutic NK cell infusions post-HSCT, and pre-emptive/prophylactic NK cell infusions post-HSCT on outcomes after allogeneic HSCT, such as survival, relapse, GVHD, and infections.

Higher NK cell recovery after HSCT was associated with favorable outcomes although studies were heterogeneous. Higher NK cell reconstitution was associated with a better 2-year OS (77% vs 55%). We could not perform pooled analysis for relapse, GVHD, or infections due to the paucity of data. A low number of NK cells after HSCT has been associated with poor outcomes and efforts to improve NK cell reconstitution have resulted in favorable responses (37, 38). Higher NK cell reconstitution has been associated with significantly lower relapse rates by Dunbar et al., Lang et al., and McCurdy et al (16, 18, 22). The early recovery of NK cells following HSCT could have antitumor effects that could explain lower relapse rates in patients with higher NK cell reconstitution.

In our meta-analysis, higher stem cell graft NK cell content was associated with a higher OS (65% vs 46.5%), lower RR (17% vs 33%), and lower acute GVHD incidence (28% vs 50%); however, the differences were not statistically significant. Improved relapse-free and overall survival were observed after HSCT with high NK cell content in the stem cell graft and early NK cell reconstitution. The rapid recovery of NK and T cells post-HSCT likely enhances the crosstalk between NK and T cells in the microenvironment (20). Our findings of lower acute GVHD in the high NK cell group are consistent with the findings of previous studies which reported that the GVL effect is possible without increasing GVHD (22, 39). This likely can be explained in part based on the restricted alloreactivity of NK cells to hematopoietic cells and partly based on innate differences between donor and host recipient’s major histocompatibility complexes (MHC)-class I profiles which are exploited by NK cells (34). The cytotoxic effects induced by the alloreactive NK cells lead to a reduction in antigen-presenting cells in the recipient and therefore, indirectly inhibit T-cell proliferation and GVHD induction (40). Some of the earlier studies suggested their role in inducing GVHD following HSCT; however, subsequent studies showed that the adoptive transfer of donor NK cells post-transplant led to a decline in the incidence of relapse and GVHD while preserving the GVL responses. This in part was attributed to the KIR-ligand mismatch on the target cells that suppresses the alloreactive T-cell responses in the recipient (41). NK cells have been shown to reduce viral reactivation (CMV and BK virus) following haploidentical transplantation (36, 42). Higher NK cell reconstitution decreases rates of CMV reactivation and improves outcomes in patients with CMV reactivation (25). Minculescu et al. reported that higher NK cell reconstitution was significantly associated with lower CMV infections (17). McCurdy et al. reported a significantly lower NRM with higher NK cell constitution (16). Our findings are consistent with prior studies reporting that a higher NK cell dose in the donor graft appears to be associated with a lower risk of relapse and improved progression-free survival (24–26, 43–45). Individual studies have shown better outcomes in patients with earlier and higher NK cell reconstitution following HSCT and poor outcomes with delayed and lower NK cell reconstitution (19, 23).

Therapeutic administration of NK cells for the relapsed hematologic disease after HSCT employed either NK cell or cytokine-induced killer (CIK) cell infusions. While both approaches yielded favorable outcomes, a few key differences were noted. Therapeutic NK cell infusions for hematologic relapse post-HSCT reported an ORR and CR of 83% and 45%, and RR, acute GVHD, and chronic GVHD rates of 52%, 20%, and 11%, respectively. Therapeutic CIK cell infusions reported an ORR and CR of 45% and 11%, and RR, acute GVHD, and chronic GVHD rates of 56%, 34.5%, and 21%, respectively. NK cell infusions appear to have better outcomes; however, a direct comparison could not be made due to the heterogeneous nature of the studies. In a previous meta-analysis to evaluate the therapeutic effect of HSCT in combination with NK cells in leukemia patients, the combination was found to decrease the incidence of GVHD after HSCT without a significant effect on OS and relapse rate (42).

Pre-emptive donor-derived NK cell infusions to prevent relapse post-HSCT had promising outcomes with one-year overall survival of 69%. The rates of overall response and complete remission were 77% and 42% respectively. The relapse rate was 28%, and acute and chronic GVHD was noted in 25% and 4% of patients, respectively. These results demonstrate significantly lower relapse rates, lower GVHD incidence, and higher survival compared to the contemporary historical population although a direct comparison is difficult given the small patient sample and different underlying hematologic malignancies (46–48). The safe and effective dose of NK cell infusion remains to be determined. It is not established whether a higher number of infused NK cells improves efficacy (31). The NK cell dose for therapeutic and pre-emptive studies varied, with a dose range of 1 x10 (5) cells/kg to 1 x10 (8) cells/kg.

The promising outcomes seen with pre-emptive studies suggest that donor-derived NK cell infusion may be a more effective prophylactic strategy as compared to NK cell infusions after overt hematologic relapse (31). Individual studies have also shown the production feasibility and safety of infusing NK cells (preemptive and therapeutic) following HSCT resulting in higher NK cell number and function, lower relapse rates, and lower viral infections without increasing GVHD or mortality (36, 45). These findings support the feasibility and potential benefit of NK cell infusions after HSCT without increasing treatment-related mortality. Further prospective studies are needed to define the optimal manipulation, expansion, dosing, and timing of NK cell infusions after HSCT. Moreover, there is emerging research on engineering NK cells for cancer immunotherapy, including chimeric antigen receptor (CAR) NK cells, that can be employed in hematologic malignancies (49, 50).

To our knowledge, this is the first comprehensive meta-analysis to investigate the impact of NK cell reconstitution and infusion on outcomes after HSCT. Our study has several limitations. Literature was limited with a lack of randomized data. Studies were very heterogeneous, limiting the generalization of results, and allowing for a limited pooled analysis. Our findings suggest the promising benefit of NK cells to prevent disease relapse without causing excess toxicity; however, these findings are hypothesis-generating and conclusions should be made with caution due to the lack of prospective randomized data.

## Conclusions

Higher reconstitution of NK cells after allogeneic HSCT appears to have a favorable impact on outcomes, including better OS and low incidence of relapse, acute GVHD, and viral infections. Infusion of donor-derived NK cells after HSCT to prevent relapse resulted in favorable outcomes with an acceptable toxicity profile. However, the benefit of allogeneic NK cell infusions after HSCT to treat overt hematologic relapse is modest. The study summarizes the current evidence regarding the impact of NK cells following allogeneic HSCT with its limitations. This seminal review will help to tailor and design phase I clinical trials evaluating unmanipulated or engineered NK cell infusions following allogeneic HSCT in patients with hematologic malignancies. Our findings suggest the need for further prospective studies to investigate the utility of NK cell infusion early post-transplant to improve clinical outcomes and survival as well as to establish the potential benefit of NK cell infusion after HSCT to prevent relapse of high-risk hematologic malignancies without increased non-relapse mortality.

## Data availability statement

The original contributions presented in the study are included in the article/**Supplementary Material**. Further inquiries can be directed to the corresponding author.

## Author contributions

All authors contributed to the manuscript and fulfilled criteria per the uniform requirements set forth by the International Committee of Medical Journal Editors (ICJME) guidelines. All authors contributed to the article and approved the submitted version.

## Conflict of interest

SC has speaking, consulting and advisory role, and research funding from in Incyte and Therakos. JM has speaking, consulting and advisory role in Kite, Juno Therapeutics, Allovir, Magenta Therapeutics, EcoR1 Capital, and has research funding from Novartis, Fresenius Biotech, Astellas Pharma, Bellicum Pharmaceuticals, Gamida Cell, Pluristem Therapeutics, Kite and AlloVir.

The remaining authors declare that the research was conducted in the absence of any commercial or financial relationships that could be construed as a potential conflict of interest.

## Publisher’s note

All claims expressed in this article are solely those of the authors and do not necessarily represent those of their affiliated organizations, or those of the publisher, the editors and the reviewers. Any product that may be evaluated in this article, or claim that may be made by its manufacturer, is not guaranteed or endorsed by the publisher.

## References

[1] Ogonek J Kralj Juric M Ghimire S Varanasi PR Holler E Greinix H . Immune reconstitution after allogeneic hematopoietic stem cell transplantation. Front Immunol (2016) 7:507. doi: 10.3389/fimmu.2016.00507 27909435 PMC5112259

[2] Almeida-Oliveira A Smith-Carvalho M Fau-Porto LC Porto LC Fau-Cardoso-Oliveira J . Age-related changes in natural killer cell receptors from childhood through old age. Hum Immunol (2011) 72(4):319–29. doi: 10.1016/j.humimm.2011.01.009 21262312

[3] Ottinger HD Beelen Dw Fau - Scheulen B Scheulen B Fau-Schaefer UW Schaefer UW Fau-Grosse-Wilde H . Improved immune reconstitution after allotransplantation of peripheral blood stem cells instead of bone marrow. Blood (1996) 88(7):2775–9. doi: 10.1182/blood.V88.7.2775.bloodjournal8872775 8839875

[4] Walzer T Jaeger S Fau - Chaix J Chaix J Fau - Vivier E Vivier E . Natural killer cells: from CD3(-)NKp46(+) to post-genomics meta-analyses. Curr Opin Immunol (2007) 19(3):365–72. doi: 10.1016/j.coi.2007.04.004 17442558

[5] De Maria A Bozzano F Fau - Cantoni C Cantoni C Fau - Moretta L Moretta L . Revisiting human natural killer cell subset function revealed cytolytic CD56(dim)CD16+ NK cells as rapid producers of abundant IFN-gamma on activation. Proc Natl Acad Sci USA (2011) 108(2):728–32. doi: 10.1073/pnas.1012356108. PMC3021076 21187373

[6] Nguyen S Kuentz M Vernant JP Dhedin N Bories D Debré P . Involvement of mature donor T cells in the NK cell reconstitution after haploidentical hematopoietic stem-cell transplantation. Leukemia (2008) 22(2):344–52. doi: 10.1038/sj.leu.2405041 18033316

[7] Kumar V McNerney ME . A new self: MHC-class-I-independent natural-killer-cell self-tolerance. Nat Rev Immunol (2005) 5(5):363–74. doi: 10.1038/nri1603 15841099

[8] Guerra N Tan YX Joncker NT Choy A Gallardo F Xiong N . NKG2D-deficient mice are defective in tumor surveillance in models of spontaneous malignancy. Immunity (2008) 28(4):571–80. doi: 10.1016/j.immuni.2008.02.016 PMC3528789 18394936

[9] Simona Sivori DP Bottino C Emanuela M Anna P Roberto B Lorenzo M . NKp46 is the major triggering receptor involved in the natural cytotoxicity of fresh or cultured human NK cells. correlation between surface density of NKp46 and natural cytotoxicity against autologous, allogeneic or xenogeneic target cells. Eur J Immunol (1999) 29(5):1656–66. doi: 10.1002/(SICI)1521-4141(199905)29:05<1656::AID-IMMU1656>3.0.CO;2-1 10359120

[10] Maddineni S Silberstein JL Sunwoo JB . Emerging NK cell therapies for cancer and the promise of next generation engineering of iPSC-derived NK cells. J ImmunoTher. Cancer. (2022) 10(5):e004693. doi: 10.1136/jitc-2022-004693 35580928 PMC9115029

[11] Chu J Gao F Yan M Zhao S Yan Z Shi B . Natural killer cells: A promising immunotherapy for cancer. J Trans Med (2022) 20(1):240. doi: 10.1186/s12967-022-03437-0 PMC9125849 35606854

[12] Ciurea SO Kongtim P Soebbing D Trikha P Behbehani G Rondon G . Decrease post-transplant relapse using donor-derived expanded NK-cells. Leukemia Jan (2022) 36(1):155–64. doi: 10.1038/s41375-021-01349-4 PMC8727305 34312462

[13] Higgins JPT Thomas J Chandler J Cumpston M Li T Page MJ . eds. Cochrane Handbook for Systematic Reviews of Interventions. 2nd Edition. Chichester (UK): John Wiley & Sons (2019).

[14] Higgins JP Thompson Sg Fau - Deeks JJ Deeks Jj Fau - Altman DG Altman DG . Measuring inconsistency in meta-analyses. BMJ (2003) 327(7414):557–60. doi: 10.1136/bmj.327.7414.557 PMC192859 12958120

[15] Higgins JP Thompson SG . Quantifying heterogeneity in a meta-analysis. Stat Med (2002) 21(11):1539–58. doi: 10.1002/sim.1186 12111919

[16] McCurdy SR Radojcic V Tsai H-L Vulic A Thompson E Ivcevic S . Signatures of GVHD and relapse after posttransplant cyclophosphamide revealed by immune profiling and machine learning. Blood (2022) 139(4):608–23. doi: 10.1182/blood.2021013054 PMC8796655 34657151

[17] Minculescu L Marquart HV Friis LS Petersen SL Schiødt I Ryder LP . Early natural killer cell reconstitution predicts overall survival in T cell-replete allogeneic hematopoietic stem cell transplantation. Biol Blood Marrow Transplant (2016) 22(12):2187–2193. doi: 10.1016/j.bbmt.2016.09.006 27664326

[18] Lang P Pfeiffer M Teltschik HM Schlegel P Feuchtinger T Ebinger M . Natural killer cell activity influences outcome after T cell depleted stem cell transplantation from matched unrelated and haploidentical donors. Best Pract Res Clin Haematol Sep (2011) 24(3):403–11. doi: 10.1016/j.beha.2011.04.009 21925093

[19] Ando T Suzuki T Ishiyama Y Koyama S Tachibana T Tanaka M . Impact of cytomegalovirus reactivation and natural killer reconstitution on outcomes after allogeneic hematopoietic stem cell transplantation: A single-center analysis. Biol Blood Marrow Transplant Jan (2020) 26(1):171–7. doi: 10.1016/j.bbmt.2019.09.028 31563574

[20] Chang YJ Zhao XY Huang XJ . Effects of the NK cell recovery on outcomes of unmanipulated haploidentical blood and marrow transplantation for patients with hematologic malignancies. Biol Blood Marrow Transplant Mar (2008) 14(3):323–34. doi: 10.1016/j.bbmt.2007.12.497 18275899

[21] Kheav VD Busson M Scieux C Peffault de Latour R Maki G Haas P . Favorable impact of natural killer cell reconstitution on chronic graft-versus-host disease and cytomegalovirus reactivation after allogeneic hematopoietic stem cell transplantation. Haematologica (2014) 99(12):1860–7. doi: 10.3324/haematol.2014.108407 PMC4258747 25085354

[22] Dunbar EM Buzzeo MP Levine JB Schold JD Meier-Kriesche HU Reddy V . The relationship between circulating natural killer cells after reduced intensity conditioning hematopoietic stem cell transplantation and relapse-free survival and graft-versus-host disease. Haematologica (2008) 93(12):1852–8. doi: 10.3324/haematol.13033 18945751

[23] Kim SY Lee H Han MS Shim H Eom HS Park B . Post-transplantation natural killer cell count: A predictor of acute graft-Versus-Host disease and survival outcomes after allogeneic hematopoietic stem cell transplantation. Clin Lymphoma Myeloma. Leuk. Sep (2016) 16(9):527–535.e2. doi: 10.1016/j.clml.2016.06.013 27375156

[24] Vela-Ojeda J García-Ruiz Esparza MA Reyes-Maldonado E Jiménez-Zamudio L García-Latorre E Moreno-Lafont M . Clinical relevance of NK, NKT, and dendritic cell dose in patients receiving G-CSF-mobilized peripheral blood allogeneic stem cell transplantation. Ann Hematol Feb (2006) 85(2):113–20. doi: 10.1007/s00277-005-0037-5 16311734

[25] Clausen J Wolf D Petzer AL Gunsilius E Schumacher P Kircher B . Impact of natural killer cell dose and donor killer-cell immunoglobulin-like receptor (KIR) genotype on outcome following human leucocyte antigen-identical haematopoietic stem cell transplantation. Clin Exp Immunol Jun (2007) 148(3):520–8. doi: 10.1111/j.1365-2249.2007.03360.x PMC1941931 17493020

[26] Kim DH Won DI Lee NY Sohn SK Suh JS Lee KB . Non-CD34+ cells, especially CD8+ cytotoxic T cells and CD56+ natural killer cells, rather than CD34 cells, predict early engraftment and better transplantation outcomes in patients with hematologic malignancies after allogeneic peripheral stem cell transplantation. Biol Blood Marrow Transplant. Jul (2006) 12(7):719–28. doi: 10.1016/j.bbmt.2006.03.005 16785061

[27] Chklovskaia E Nowbakht P Nissen C Gratwohl A Bargetzi M Wodnar-Filipowicz A . Reconstitution of dendritic and natural killer-cell subsets after allogeneic stem cell transplantation: effects of endogenous flt3 ligand. Blood (2004) 103(10):3860–8. doi: 10.1182/blood-2003-04-1200 14764540

[28] Introna M Borleri G Conti E Franceschetti M Barbui AM Broady R . Repeated infusions of donor-derived cytokine-induced killer cells in patients relapsing after allogeneic stem cell transplantation: A phase I study. Haematologica. Jul (2007) 92(7):952–9. doi: 10.3324/haematol.11132 17606446

[29] Laport GG Sheehan K Baker J Armstrong R Wong RM Lowsky R . Adoptive immunotherapy with cytokine-induced killer cells for patients with relapsed hematologic malignancies after allogeneic hematopoietic cell transplantation. Biol Blood Marrow Transplant (2011) 17(11):1679–87. doi: 10.1016/j.bbmt.2011.05.012 PMC3175285 21664472

[30] Linn YC Niam M Chu S Choong A Yong HX Heng KK . The anti-tumour activity of allogeneic cytokine-induced killer cells in patients who relapse after allogeneic transplant for haematological malignancies. Bone Marrow Transplant (2012) 47(7):957–66. doi: 10.1038/bmt.2011.202 21986635

[31] Shaffer BC Le Luduec JB Forlenza C Jakubowski AA Perales MA Young JW . Phase II study of haploidentical natural killer cell infusion for treatment of relapsed or persistent myeloid malignancies following allogeneic hematopoietic cell transplantation. Biol Blood Marrow Transplant (2016) 22(4):705–9. doi: 10.1016/j.bbmt.2015.12.028 PMC4801764 26772158

[32] Lee DA Denman CJ Rondon G Woodworth G Chen J Fisher T . Haploidentical natural killer cells infused before allogeneic stem cell transplantation for myeloid malignancies: A phase I trial. Biol Blood Marrow Transplant Jul (2016) 22(7):1290–8. doi: 10.1016/j.bbmt.2016.04.009 PMC4905771 27090958

[33] Stern M Passweg JR Meyer-Monard S Esser R Tonn T Soerensen J . Pre-emptive immunotherapy with purified natural killer cells after haploidentical SCT: A prospective phase II study in two centers. Bone Marrow Transplant Mar (2013) 48(3):433–8. doi: 10.1038/bmt.2012.162 22941380

[34] Passweg JR Tichelli A Meyer-Monard S Heim D Stern M Kühne T . Purified donor NK-lymphocyte infusion to consolidate engraftment after haploidentical stem cell transplantation. Leukemia. Nov (2004) 18(11):1835–8. doi: 10.1038/sj.leu.2403524 15457184

[35] Choi I Yoon SR Park SY Kim H Jung SJ Jang YJ . Donor-derived natural killer cells infused after human leukocyte antigen-haploidentical hematopoietic cell transplantation: A dose-escalation study. Biol Blood Marrow Transplant (2014) 20(5):696–704. doi: 10.1016/j.bbmt.2014.01.031 24525278

[36] Ciurea SO Schafer JR Bassett R Denman CJ Cao K Willis D . Phase 1 clinical trial using mbIL21 ex vivo-expanded donor-derived NK cells after haploidentical transplantation. Blood (2017) 130(16):1857–68. doi: 10.1182/blood-2017-05-785659 PMC5649552 28835441

[37] Hattori N Saito B Sasaki Y Shimada S Murai S Abe M . Status of natural killer cell recovery in day 21 bone marrow after allogeneic hematopoietic stem cell transplantation predicts clinical outcome. Biol Blood Marrow Transplant (2018) 24(9):1841–7. doi: 10.1016/j.bbmt.2018.05.007 29753837

[38] Alvarez MA-O Dunai CA-O Khuat LT Aguilar EG Barao I Murphy WJ . IL-2 and anti-TGF-β promote NK cell reconstitution and anti-tumor effects after syngeneic hematopoietic stem cell transplantation. Cancers (Basel) (2020) 12(11):3189. doi: 10.3390/cancers12113189 PMC7692743 33138229

[39] Ruggeri L Capanni M Urbani E Perruccio K Shlomchik WD Tosti A . Effectiveness of donor natural killer cell alloreactivity in mismatched hematopoietic transplants. Science (2002) 295(5562):2097–100. doi: 10.1126/science.1068440 11896281

[40] Meinhardt K Kroeger I Bauer R Ganss F Ovsiy I Rothamer J . Identification and characterization of the specific murine NK cell subset supporting graft-versus-leukemia- and reducing graft-versus-host-effects. Oncoimmunol. Jan (2015) 4(1):e981483. doi: 10.4161/2162402x.2014.981483 PMC4368119 25949862

[41] Leung W . Use of NK cell activity in cure by transplant. Br J Haematol (2011) 155(1):14–29. doi: 10.1111/j.1365-2141.2011.08823.x 21812770

[42] Zhang P Yang S Zou Y Yan X Wu H Zhou M . NK cell predicts the severity of acute graft-versus-host disease in patients after allogeneic stem cell transplantation using antithymocyte globulin (ATG) in pretreatment scheme. BMC Immunol Dec 9 (2019) 20(1):46. doi: 10.1186/s12865-019-0326-8 PMC6902350 31818250

[43] Rubio MT Moreira-Teixeira L Bachy E Bouillié M Milpied P Coman T . Early posttransplantation donor-derived invariant natural killer T-cell recovery predicts the occurrence of acute graft-versus-host disease and overall survival. Blood (2012) 120(10):2144–54. doi: 10.1182/blood-2012-01-404673 22730537

[44] Malard F Labopin M Chevallier P Guillaume T Duquesne A Rialland F . Larger number of invariant natural killer T cells in PBSC allografts correlates with improved GVHD-free and progression-free survival Blood (2016) 127(14):1828–35. doi: 10.1182/blood-2015-12-688739 26903546

[45] de Lalla C Rinaldi A Montagna D Fau-Azzimonti L Bernardo ME Sangalli LM . Invariant NKT cell reconstitution in pediatric leukemia patients given HLA-haploidentical stem cell transplantation defines distinct CD4+ and CD4- subset dynamics and correlates with remission state. J Immunol (2011) 186(7):4490–9. doi: 10.4049/jimmunol.1003748 21357532

[46] Koreth J Schlenk R Kopecky KJ Honda S Sierra J Djulbegovic BJ . Allogeneic stem cell transplantation for acute myeloid leukemia in first complete remission: systematic review and meta-analysis of prospective clinical trials. JAMA (2009) 301(22):2349–61. doi: 10.1001/jama.2009.813 PMC3163846 19509382

[47] Gomez-Arteaga A Gyurkocza B . Recent advances in allogeneic hematopoietic cell transplantation for acute myeloid leukemia. Curr Opin Hematol Mar (2020) 27(2):115–21. doi: 10.1097/moh.0000000000000572 31913152

[48] Evers G Beelen DW Braess J Sauerland C Kolb H-J Reichle A . Outcome of patients with acute myeloid leukemia (AML) undergoing allogeneic hematopoietic stem cell transplantation (HSCT) beyond first complete remission (CR1). Blood (2018) 132(Suppl 1):4649–9. doi: 10.1182/blood-2018-99-116964

[49] Schmidt P Raftery MJ Pecher G . Engineering NK cells for CAR therapy-recent advances in gene transfer methodology. Front Immunol (2020) 11:611163. doi: 10.3389/fimmu.2020.611163 33488617 PMC7817882

[50] Liu S Galat V Galat Y Lee YKA Wainwright D Wu J . NK cell-based cancer immunotherapy: from basic biology to clinical development. J Hematol Oncol (2021) 14(1):7. doi: 10.1186/s13045-020-01014-w 33407739 PMC7788999

