# Supplementary material for: Impact of natural killer cells on outcomes after allogeneic hematopoietic stem cell transplantation: A systematic review and meta-analysis
Source: Front Immunol. 2022 Oct 3;13:1005031. doi: 10.3389/fimmu.2022.1005031 (PMC9574024; doi:10.3389/fimmu.2022.1005031)
Supplement: Supplementary file 1 [file Table_1.docx]

Supplementary Material

**Supplementary Table 1: Excluded studies in second screening with reason and DOI**

| **No.** | **Author Name** | **Reason For Exclusion** | **DOI/Link/Reference** |
| --- | --- | --- | --- |
| 1 | Nowak et al. | Irrelevant | DOI: 10.1002/ajh.23802 |
| 2 | Pical-Izzard et al. | Irrelevant | DOI: 10.1016/j.bbmt.2014.11.681 |
| 3 | Thakar et al. | No Full Article | DOI: 10.1182/blood.V128.22.1161.1161 |
| 4 | Zhang et al. | Focuses on GVHD | DOI: 10.1186/s12865-019-0326-8 |
| 5 | Beelen et al. | Irrelevant | DOI: 10.1182/blood-2004-04-1441 |
| 6 | MD Anderson Trial | Irrelevant | https://ClinicalTrials.gov/show/NCT00536978 |
| 7 | MSK trial | Irrelevant | https://ClinicalTrials.gov/show/NCT00526292 |
| 8 | Masonic Cancer Center Trial | Irrelevant | https://ClinicalTrials.gov/show/NCT00303667 |
| 9 | Ureshino et al. | Case Report | DOI: 10.1007/s12185-019-02809-5 |
| 10 | Park et al. | Pediatric | DOI: 10.1111/ctr.13147 |
| 11 | Jaiswal et al. | Pediatric | DOI: 10.1016/j.jcyt.2016.12.006 |
| 12 | Jaiswal et al. | Special Report | DOI: 10.2217/imt-2019-0037 |
| 13 | Goh et al. | Pediatric | DOI: 10.5045/kjh.2011.46.1.18 |
| 14 | Bachanova et al. | Not full article | DOI: 10.1016/j.bbmt.2015.01.008 |
| 15 | De Witte et al. | Irrelevant | DOI: 10.1016/j.bbmt.2018.02.023 |
| 16 | Foley et al. | Irrelevant | DOI: 10.1182/blood-2011-04-347070 |
| 17 | Ghasemzadeh et al. | Irrelevant | DOI: 10.1016/j.leukres.2015.12.002 |
| 18 | Roberto et al. | Irrelevant | DOI: 10.3324/haematol.2017.186619 |
| 19 | Gartner et al. | Focuses on GVHD | DOI: 10.1016/j.jcyt.2013.05.016 |
| 20 | Rubio et al. | Irrelevant | DOI: 10.1182/blood-2012-01-404673 |
| 21 | Peterson et al. | Irrelevant | DOI: 10.1038/sj.bmt.1704084 |
| 22 | Giebel et al. | Irrelevant | https://pubmed.ncbi.nlm.nih.gov/16218030/ |
| 23 | Giebel et al. | Irrelevant | DOI: 10.1016/j.transproceed.2005.11.091 |
| 24 | Ruggeri et al. | Review Article | DOI: 10.1016/j.semcancer.2006.07.007 |
| 25 | Hutchinson et al. | Irrelevant | https://clinicaltrials.gov/ct2/show/NCT00112593 |
| 26 | Penack et al. | Irrelevant | DOI: 10.1038/sj.bmt.1705911 |
| 27 | Ruggeri et al. | Chapter in book | DOI: 10.1007/978-0-387-78580-6_3 |
| 28 | Yu et al. | Chinese | https://pubmed.ncbi.nlm.nih.gov/19236771/ |
| 29 | De Angelis et al. | Irrelevant | DOI: 10.1159/000323661 |
| 30 | Federman et al. | Irrelevant | DOI: 10.1038/leu.2010.235 |
| 31 | Vago et al. | Irrelevant | DOI: 10.1182/blood-2007-07-103325 |
| 32 | Giebel et al. | Irrelevant | DOI: 10.1038/bmt.2009.384 |
| 33 | Pittari et al. | Irrelevant | DOI: 10.1038/bmt.2009.265 |
| 34 | Tripplet et al. | Case Report | DOI: 10.1016/s0145-2126(02)00090-5 |
| 35 | Scholl et al. | Irrelevant | DOI: 10.1038/sj.bmt.1704752 |
| 36 | Meerim et al. | Irrelevant | DOI: 10.1111/ctr.13147 |
| 37 | Chaidos et al. | Irrelevant | DOI: 10.1182/blood-2011-11-389304. |
| 38 | Rubio et al. | Irrelevant | DOI: 10.1182/blood-2012-01-404673 |
